# Bile Acids in Inflammatory Bowel Disease: From Pathophysiology to Treatment

**DOI:** 10.3390/biomedicines12122910

**Published:** 2024-12-20

**Authors:** Samantha H. Bai, Arun Chandnani, Siyan Cao

**Affiliations:** Division of Gastroenterology, Department of Medicine, Washington University School of Medicine, St. Louis, MO 63110, USA; s.bai@wustl.edu (S.H.B.); arunc@wustl.edu (A.C.)

**Keywords:** bile acids, inflammatory bowel disease, ulcerative colitis, Crohn’s disease, pathogenesis, new therapies

## Abstract

Inflammatory bowel disease (IBD) is a chronic condition that affects about 7 million people worldwide, and new therapies are needed. Understanding the complex roles that bile acids (BAs) play in IBD may lead to the development of novel IBD treatments independent of direct immunosuppression. This review discusses the latest discoveries in the roles BAs play in IBD pathogenesis and explores how these discoveries offer promising new therapeutic targets to treat IBD and improve patient outcomes. Several therapies discussed include specific BA receptor (BAR) agonists, dietary therapies, supplements, probiotics, and mesenchymal stem cell therapies that have all been shown to decrease IBD disease activity.

## 1. Introduction

Inflammatory bowel disease (IBD), which includes ulcerative colitis (UC) and Crohn’s disease (CD), is a chronic, relapsing disease that affects about 7 million people worldwide [1,2]. IBD is a multifactorial disease with complex interactions between the diet, genetics, environment, gut microbiome, and the immune system, although the mechanisms remain poorly understood [3]. Current IBD treatments include biological and small molecule therapies that target the immune system. These medications can have significant side effects, including infection, malignancy, and thromboembolism. In addition, they only achieve sustained remission in a subset of patients [4], underscoring the need for new therapeutic approaches.

Recent advances have shown that gut metabolites such as bile acids (BAs) are also critical in IBD. BAs are amphipathic molecules derived from cholesterol to form primary BAs. These primary BAs undergo enterohepatic cycling and can be deconjugated by the gut microbiota to form secondary BAs. BAs exert their effects on the intestine by operating as signaling molecules that activate a variety of BA receptors (BARs), thereby regulating intestinal homeostasis [5].

A deeper understanding of the role of BAs in IBD may identify previously unknown pathogenesis and unveil novel therapies for IBD. In this review, we provide a comprehensive overview of the most recent studies elucidating the synthesis and function of BAs and their multifactorial roles in IBD pathogenesis, discuss several potential BA-based treatments for IBD, and identify areas of further research to advance our understanding of the complex interplay between BAs and IBD.

A comprehensive search of the PubMed, Embase, and Scopus databases from January 2020 until October 2024 was conducted to identify English-language articles regarding the role of bile acids in IBD. The specific search terms employed were the following: “bile acid”, “bile salt”, “Inflammatory Bowel Diseases”, “IBD”, “Crohn’s disease”, “CD”, “ulcerative colitis”, “UC”, and “colitis”. The screening process involved two independent reviewers (S.H.B. and S.C.) who first assessed titles and abstracts to identify potentially relevant studies. The full texts of these articles were examined to determine if they were eligible for inclusion as well as their reference lists to ensure relevant studies were included. The ultimate decision to include abstracts and articles was based on their relevance to the research objectives.

## 2. Overview of BAs and Their Emerging Roles in IBD

### 2.1. Synthesis of BAs

BAs are amphiphilic steroid molecules that play important roles in a variety of physiological processes, including dietary absorption of lipids and vitamins, protection against microbes, and regulation of the gut microbiota and intestinal inflammation [3,6,7,8]. Primary BAs are derived from cholesterol in the liver via two pathways: classic and alternative pathways [9,10]. Via a series of cytochrome P450 enzymes, cholesterol is converted to cholic acid (CA) or chenodeoxycholic acid (CDCA) in the classical pathway and CDCA in the alternative pathway [11]. CA and CDCA are the most common primary BAs in humans, while mice and infants generate 6-hydroxylated versions of CA and CDCA, known as muricholic acid (MCA) [12,13,14,15]. CA and CDCA are subsequently conjugated with glycine or taurine via BA-CoA synthase and BA-CoA amino acid N-acetyltransferase [16,17]. The conjugated BAs are secreted from the liver to the gallbladder and released into the duodenum after meals [18]. In the small intestine, 95% of BAs are reabsorbed in the ileum via the apical sodium-dependent bile acid transporter (ASBT) and returned to the liver via the portal vein [19,20,21]. This enterohepatic circulation occurs between 4 and 12 times per day in humans [22,23]. The remaining 5% of BAs that are not reabsorbed enter the colon, where they are excreted in the feces and undergo biotransformation by the intestinal microbiota to form secondary BAs [24,25,26]. The main microbial biotransformation reactions include deconjugation by bile salt hydrolases (BSHs), 7α/β-dehydroxylation by bacterial BA-inducible (*bai*) genes, and oxidation and epimerization by pyridine nucleotide-dependent hydroxysteroid dehydrogenases (HSDHs) [17,27,28]. The two major secondary BAs in humans are lithocholic acid (LCA) and deoxycholic acid (DCA), which are produced from CDCA and CA, respectively [29,30,31]. 

BAs exert their biological effects primarily by serving as ligands for BARs [32]. The main BARs are the nuclear receptors farnesoid X receptor (FXR), retinoid-related orphan receptor γt (RORγt), vitamin D receptor (VDR), pregnane X receptor (PXR), and constitutive androstane receptor (CAR), as well as the membrane-bound receptors G protein-coupled BAR 1, also known as Takeda G protein-coupled receptor 5, (GPBAR1 or TGR5), and sphingosine-1-phosphate receptor 2 (S1PR2) [5,33,34]. BAs also regulate BA synthesis via feedback mechanisms. The BA pool that returns to the liver from the ileum directly suppresses the production of new BAs in hepatocytes via FXR, which increases fibroblast growth factor expression (FGF-15 in mice and FGF-19 in humans) and suppresses BA synthesis [35,36]. Understanding these complex interactions and feedback mechanisms is crucial for elucidating how BAs influence the development of IBD.

### 2.2. BA Alterations in Patients with IBD

Patients with IBD develop an altered BA composition [37] characterized by an increase in primary BAs and a decrease in secondary BAs [38,39,40]. While most studies were conducted on adult patients with IBD, similar trends have been found in pediatric patients [41]. Recent analyses of pooled samples of patients with CD and UC revealed an increase in the primary BA glycocholic acid (GCA) [37] and decreases in the secondary BAs DCA [38], LCA [38], glycodeoxycholic acid (GDCA) [38], glycolithocholic acid (GLCA) [38], isolithocholic acid (isoLCA) [39], 3-oxolithocholic acid (3-oxoLCA) [39], taurodeoxycholic acid (TDCA) [38], and taurolithocholic acid (TLCA) [38]. The reduction in secondary BAs is thought to result from gut dysbiosis in IBD patients, which reduces primary BA deconjugation and 7α-dehydroxylation by the gut microbiota, leading to the depletion of secondary BAs [40,41]. However, there is some conflicting data as another study showed that both UC and CD patients had a decrease in the primary BA CDCA [38]. Both CD [42,43] and UC [42] patients also had increases in the total fecal BAs compared to healthy controls. It remains to be determined whether disease duration and activity, location of inflammation, and past and current treatments affect the BA pool in IBD patients. 

Patients with CD showed increased levels of the primary BAs CA [44,45], CDCA [44], and GCA [46,47], and decreased levels of the secondary BAs DCA [48,49] and LCA [46,48,49]. Secondary BAs were even lower in patients with active CD compared to patients with CD in remission [48]. Yet other studies have found decreased levels of the primary BAs glycochenodeoxycholic acid (GCDCA) [46], glycohyodeoxycholic acid (GHDCA) [50], and taurocholic acid (TCA) [46], and increased levels of the secondary BAs DCA [47,51], GDCA [46,47], isoLCA [47], and TDCA [47] in CD patients. Patients with ileal CD exhibited increased levels of primary BAs and secondary BAs compared to those with colonic CD [52]. Surgeries have also been shown to skew BA species in the intestine. Compared to CD patients who did not undergo surgery, CD patients who underwent ileal resection had more CA [53] and less isoLCA [54], although other studies found an increase in DCA [53]. These changes in BAs with ileal resection are thought to be due to alterations in the gut microbiota [54]. CD patients who underwent ileocecectomy were more susceptible to ileitis and those who developed ileitis exhibited elevated CA and CDCA levels, reduced microbial diversity, and decreased abundance of *Faecalibacterium prausnitzii* compared to those without ileitis [53]. In addition to intestinal inflammation, altered BAs have been linked to psychological disorders in CD, although the underlying mechanisms are unknown. The self-rated depression scale positively correlated with 7-dehydrocholic acid (7-DHCA) and negatively correlated with hyodeoxycholic acid (HDCA) and 12-DHCA, while the self-rated anxiety scale positively correlated with TDCA, TLCA, and tauro-β-MCA (TβMCA) [49]. 

Similarly, UC patients have also exhibited an altered BA composition in the digestive tract, such as increased levels in the primary BAs CA [55,56], CDCA [56], GCA [56], GCDCA [55,57], TCA [55,56], and taurochenodeoxycholic acid (TCDCA) [55,57], and decreased levels in the secondary BAs DCA [50,55,56], LCA [50,55,56], GDCA [55], GLCA [55], HDCA [56], isoLCA [56], 12-ketolithocholic acid (12-KLCA) [56], and TLCA [55]. Patients with more severe UC exhibited greater decreases in secondary BAs than those with less severe UC [56,58]. Ethnicity may also influence BA composition as Hispanics and South Asians with UC have higher levels of primary BAs [59] and LCA [60] than Caucasians. UC patients who underwent a total proctocolectomy with ileal pouch-anal anastomosis (IPAA) had lower levels of DCA and LCA [61], as well as decreased secondary BA-producing bacteria, such as Ruminococcaceae and those with *bai* genes, further suggesting that gut dysbiosis in IBD patients leads to secondary BA deficiency [62]. BAs may also explain the differential effects of smoking on UC versus CD. The 3-beta-hydroxysteroid dehydrogenase enzyme, which is involved in BA metabolism, was upregulated in CD patients who never smoked and was downregulated in UC patients who currently smoke [63]. In support of the connection between altered BAs and psychological disorders in UC, UC patients with high-stress reactivity had decreased levels of CDCA, GCA, GCDCA, and LCA than those with lower stress reactivity [64].

While there is a general association between increased primary BAs and decreased secondary BAs in IBD patients, some studies have conflicting results regarding specific BAs (Table 1), which raises concerns regarding the roles specific BAs play in IBD pathogenesis. However, several key differences between studies may contribute to the contradictory results. For example, one study measured BAs in fecal samples, while another used serum samples [43,49]. Differences in patient populations and sample collection timings may also contribute to these results. 

In addition to intestinal inflammation, BA dysregulation may contribute to higher risks of primary sclerosing cholangitis (PSC) and colorectal cancer in IBD patients. IBD-PSC patients exhibited decreased sulfated BAs, increased conjugated secondary BAs, and elevated liver fibrosis compared to IBD-only patients [65]. This may be partly due to differences in the gut microbiota as PSC-IBD patients exhibited increased microbial genes involved in secondary BA metabolism with enrichment in *Veillonella atypica*, *Veillonella dispar*, and *Clostridium scindens* [65]. Patients with UC and PSC who underwent IPAA and later developed chronic pouchitis exhibited lower levels of LCA and decreased microbiota α-diversity than those with a normal pouch [66]. Abnormal BA metabolism and gut dysbiosis may also contribute to colitis-associated cancer. Mice with azoxymethane (AOM) and dextran sulfate sodium (DSS)-induced colon cancer exhibited a reduction in *Clostridium XlV* and *Lactobacillus*, decreased secondary BAs, downregulation of the FXR-FGF15 axis, and upregulation of TGR5 [67].

### 2.3. Roles of BAs in IBD Pathogenesis

**Figure 1:**
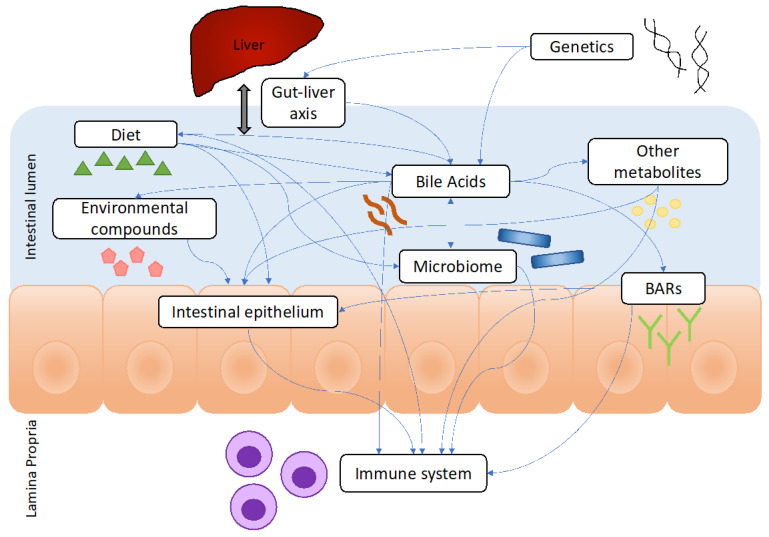
Overview of complex interactions that BAs have with factors that contribute to IBD. BAs contribute to IBD pathogenesis through numerous interactions displayed by arrows with the diet, environment, genetics, gut–liver axis, microbiome, other metabolites, BARs, intestinal epithelium, and the immune system. The figure shows several ways that BAs interact with various factors that contribute to IBD. IBD: inflammatory bowel disease; BAR: bile acid receptor.

Studies in the past decade suggest that BAs impact the pathogenesis of IBD through dysregulation of the gut microbiome, immune system, BARs, intestinal epithelium, gut–liver axis, diet, environment, and other metabolites (Figure 1). IBD patients are known to develop gut dysbiosis, which may contribute to the BA changes in IBD by skewing BA metabolism [68]. BSH gene abundance was associated with CD and UC [69]. IBD patients exhibited reductions in BA-related genes, including *cbh* and *baiN* in the gut microbiota, especially in *Alistipes* [70], *Blautia* [70], *Butyricicoccus* [55], *Clostridium IV* [55], *Clostridium XlV* [55], *Collinsella* [70], *Faecalibacterium* [55,70], and *Roseburia* [55]. Several studies in animal models of IBD also demonstrated that a shift in BAs correlated with changes in the gut microbiome. *Fusobacterium nucleatum* worsened DSS-induced colitis, partially by dysregulating BA metabolism [71]. Yorkshire pigs with DSS-induced colitis exhibited decreased BAs, possibly due to increases in *Bilophila* and *Alistipes* [72]. Yorkshire Terrier dogs with chronic enteropathy exhibited reduced UDCA as well as *Fusobacterium* and *Clostridium Hiranonis* compared to healthy controls, which only partially improved with clinical remission [73]. CD patients with the manganese transporter ZIP8 A391T mutation, which increases the risk of CD, exhibited lower levels of *Veillonella* and FGF19, suggesting that ZIP8 alters the gut microbiome and BA metabolism [74]. Microbial biofilms may also play an important role in the BA regulation of IBD as UC patients with mucosal biofilms had less bacterial diversity and more primary BAs compared to those without biofilms [75]. In addition, mucous-colonizing bacteria in IBD patients positively correlated with endoplasmic reticulum (ER) stress-related BA signals [76]. Overall, the studies suggest that there are identifiable microbial genetic differences involved in BA metabolism that are found in the gut microbiota of patients with IBD and that there are specific bacteria involved in BA metabolism that are associated with IBD. This suggests that microbiota-targeted therapies that modulate BA metabolism may be potential IBD treatments. However, there is variability in several factors between the aforementioned studies, such as varying diets and environments of the respective patient populations, which may affect the composition of the gut microbiome. Thus, it remains unclear if there are specific bacteria or genetic differences that are most implicated in IBD pathogenesis, and more studies are needed to further explore this relationship.

Modulating the immune system is another key role BAs may play in IBD pathogenesis [77]. Primary BAs are generally pro-inflammatory while secondary BAs have anti-inflammatory properties. In UC patients, the primary BAs GCA, GCDCA, and TCA positively correlated with IL-1α and TNF-α while the secondary BAs DCA, GDCA, LCA, and TLCA negatively correlated with these pro-inflammatory cytokines [55]. BAs also regulate specific immune cell subsets that control intestinal homeostasis. One good example is the T_H_17-T_reg_ cell balance, which is essential for mucosal homeostasis [78]. For example, 3-oxoLCA bound to RORγt, leading to the suppression of T_H_17 cell differentiation, while isoallolithocholic acid (isoalloLCA) promoted the differentiation of T_reg_ cells through FOXP3 induction mediated by mitochondrial reactive oxygen species [79]. The primary BA CA was increased by an inulin fiber diet, leading to IL-33 upregulation and worsening DSS-induced murine colitis and human IBD, which are likely mediated by group 2 innate lymphoid cells (ILC2s) and eosinophils [80]. In DSS-induced colitis, CA, CDCA, and LCA regulated RORγt^+^ T_reg_ levels via VDR [81], and UDCA, isoalloLCA, and 3-oxoLCA increased the proportion of ILC3s in the intestine [82], thereby improving epithelial barrier integrity and alleviating colitis. Other metabolites, including short-chain fatty acids, also work with the immune system to regulate BAs; for example, tumor necrosis factor (TNF-α), interferon-γ (IFN-γ), and butyrate-inhibited TCA uptake by Caco-2 cells, possibly through the PI3K and JAK/STAT1 pathways [83]. 

BA signaling via BARs is likely another important regulator of IBD [84]. Pediatric IBD patients exhibited reduced expression of FXR, PXR, and TGR5 [85]. UC patients exhibited increased TGR5 and decreased VDR compared to healthy controls [55]. FXR and PXR were less activated in CD patients [46]. In addition, mice with *Tgr5* disruption in intestinal stem cells developed more severe DSS-induced colitis than wild-type mice [86]. Genetics, including mutations in BAR genes, have also been shown to affect BA metabolism and IBD. The *FXR-1GT* single nucleotide variation, which leads to decreased activation of FXR targets, is associated with earlier onset and more frequent surgeries in women with CD, possibly through estrogen-mediated ER attenuation of FXR activation [87]. BARs, including FXR, PXR, VDR, and GPBAR1, are highly expressed in innate and adaptive immune cells that impact IBD pathophysiology [79,88,89,90,91,92,93], e.g., TGR5R-dependent protein kinase A activation drove the ubiquitination and phosphorylation of NLRP3, a key regulator of innate immune response in the intestinal mucosa. BAs and TGR5 activation were shown to inhibit NLRP3 inflammasome activation and inflammation in vivo [90]. A later study revealed that FXR directly interacted with NLRP3 and caspase 1, leading to the suppression of NLRP3 inflammasome. Consequently, *Fxr^−/−^* mice were more susceptible to endotoxemia shock [91]. BARs also control lymphocyte activation in inflammation. CXCR6^+^ natural killer T (NKT) cells mediated tumor suppression in the liver, which relied on the primary BA-to-secondary BA conversion by the intestinal microbiome [94]. Future studies should determine how the BAR-regulated immune responses orchestrate IBD pathogenesis. 

Impaired gut barrier function is an essential contributing factor to IBD. The regulation of intestinal epithelial tight junctions by BAs may also impact the development of IBD. DCA has been shown to increase the mRNA expression of occludin and zonula occludens-1 (ZO-1) in tight junctions, which are essential for preserving the integrity of tight junctions [95]. Knocking out *Fxr* in mice improved the intestinal barrier through improved maintenance of tight junctions and downregulated inflammatory cytokines, leading to decreased intestinal inflammation in several mouse models of colitis [96]. 

The gut–liver axis may play a significant role in the BA modulation of IBD. IBD patients have exhibited higher levels of hepatic FGF19, an inhibitor of BA production, during remission than in active disease [97]. The hepatic RelA/STAT3-CYP enzyme pathway increased primary BAs and exacerbated DSS-induced colitis [98]. Knocking out the gene encoding for G protein guanine nucleotide-binding protein subunit alpha 13 in the liver reversed the increases in CA and CDCA by upregulating the BA transporter ABCB11 in mice with DSS-induced colitis, further demonstrating that hepatic modulation of BAs is important for IBD pathogenesis [99]. BAs are also key modulators in patients with both IBD and liver disease. PSC-UC patients exhibited elevated BA signaling pathways and altered microbiota compared to patients with UC alone [100]. Mice with DSS-induced colitis developed metabolic dysfunction-associated steatotic liver disease (MASLD), partly due to BA dysregulation [101]. Cholestatic liver disease was also attenuated by suppressing BA synthesis in an IBD-PSC model with *Mdr2^−/−^* mice treated with DSS [102]. In two mouse models of MASLD and colitis, MASLD induced gut dysbiosis and increased secondary BAs in the ileum, leading to ileitis via CD8^+^ T cells and the TGR5/mTOR/oxidative phosphorylation signaling pathway [103]. Ileitis then inhibited hepatic FXR activation and worsened MASLD, suggesting that secondary BAs may be a critical link between MASLD and IBD [103]. 

The interplay between BAs and diet may be another crucial regulator of IBD. Mice with active colitis on a high-fat diet exhibited increased ferroptosis compared to those with a normal diet, which was likely caused by increased DCA levels driven by upregulated hypoxia-inducible factor-2α and divalent metal transporter-1 [104]. Multiple studies have demonstrated that western-style diets worsen DSS- and 2,4,6-trinitrobenzene sulfonic acid (TNBS)-induced colitis by dysregulating BA metabolism, including increasing DCA and LCA and decreasing the spatial distribution of TCA. A high-fat/high-sugar maternal diet elevated offspring susceptibility to TNBS-induced colitis by increasing Bacteroidetes, thereby promoting gasdermin D (GSDMD)-mediated pyroptosis and IL-1β production in macrophages [105]. Transition feeding of a high-fat diet induced BA accumulation in the ER by TNF-α and exacerbated intestinal epithelial apoptosis via the activation of the ER stress sensor IRE1α [106]. Those diets were also shown to skew macrophages toward M1 polarization and pro-inflammatory cytokine production [107], and decrease *Clostridium scindens* and secondary Bas, including isoalloLCA, leading to dysregulation of T_H_17/T_reg_ cells [108]. Moreover, a ketogenic diet, which is also high in fats, also worsened DSS-induced colitis, partly by increasing BAs, including CA, GCA, and TCDCA [109]. A study by Liu et al. found that Paneth cell dysregulation, which contributes to CD, was induced by a Western-style diet via upregulation of DCA by *baiCD*-expressing *Clostridium* spp., which increased FXR activation and type I IFNs [110]. In contrast, another study showed that a lard-based high-fat diet protected against DSS-induced colitis and colitis-associated cancer by increasing gut microbial diversity, including Firmicutes and *Clostridium* cluster XIVa abundance, upregulating secondary BAs and modulating the VDR pathway [111]. This discrepancy may be because these studies used high-fat diets from different manufacturers, which have different compositions. 

IBD activity may also be modulated by interactions between BAs, the environment, and other metabolites. One study suggested that UC patients exhibited increased environmental exposures to perfluoroalkyl substance perfluorooctanoic acid compared to CD patients and controls [112]. This exposure may contribute to colitis by altering the intestinal barrier, decreasing LCA and DCA, altering the T_H_17/T_reg_ balance [113], increasing IL-17A, and downregulating IL-10 [112,113]. Aflatoxin B_1_ exposure decreased BAs, including CDCA, and induced a similar gut dysbiosis in rats to those found in IBD patients [114]. BAs may also modulate IBD by altering short-chain fatty acid metabolism. Butyrate is downregulated in IBD, which may be partly due to DCA and CDCA inhibition of butyrate uptake [115]. These findings highlight the intricate relationship between BAs and IBD through interactions with the environment, gut metabolome, diet, genetics, gut–liver axis, intestinal epithelium, BARs, immune system, and the microbiome. This dynamic interplay has revealed several potential clinical applications for BAs in IBD, and further studies are needed to facilitate the development of novel BA-based IBD therapies. 

### 2.4. BAs as Biomarkers of IBD

Studies have suggested that BAs may serve as biomarkers of IBD disease activity and treatment response. Elevated fecal calprotectin, elevated C-reactive protein (CRP), and diarrhea correlated with BA levels in UC but not CD patients [50]. Specifically, elevated fecal calprotectin was associated with decreased levels of GLCA, and HDCA; increased CRP correlated with decreases in DCA, TDCA, LCA, TLCA, ursodeoxycholic acid (UDCA), and HDCA; and worsening diarrhea was associated with decreased levels of DCA, GDCA, and LCA [50]. The ratio of primary BAs to secondary BAs also stratified IBD activity, further demonstrating that BAs may be potential biomarkers for IBD activity [116].

BAs may be used to predict and monitor response to IBD treatment. Pediatric CD patients who achieved and sustained remission with exclusive enteral nutrition (EEN) exhibited increased secondary BAs (DCA and LCA), while those who did not sustain remission exhibited increased primary BAs (CA and CDCA) [117]. UC patients who responded to mesalamine had more secondary BAs, especially 12-KLCA [118]. The enrichment of GLCA, GDCA, and UDCA, as well as the abundance of microbial *bai* genes, predicted early remission in IBD patients undergoing anti-cytokine therapy [119]. Non-responsiveness to anti-TNFs was associated with higher levels of sulfate- and glycine-conjugated primary BAs in CD patients [120] and elevated CA in UC patients [106]. CD patients who responded to anti-α4β7 integrin therapy exhibited elevated secondary BAs, including LCA and DCA [121,122]. In addition to stratifying IBD severity and therapeutic response, BAs have been identified as markers in IBD patients for BA malabsorption [123], more aggressive PSC [65], and poorer response to anti-SARS-CoV-2 vaccination [124]. BAs may also serve as response biomarkers for fecal microbiota transplantation (FMT) in UC, as patients with active UC who achieved clinical response and remission with FMT showed an increase in primary BA biosynthesis [125], while another study showed that FMT restored secondary BAs [126]. Children with IBD and *Clostridioides difficile* infection (CDI) treated with FMT normalized their BAs to donor levels with a decrease in primary BAs and an increase in secondary BAs 6 months post-FMT [127]. These studies underscore the potential of BAs as valuable tools that clinicians can use to manage IBD by serving as promising biomarkers for IBD disease activity, treatment choice, and management of complications.

### 2.5. BA-Based IBD Therapies

**Figure 2:**
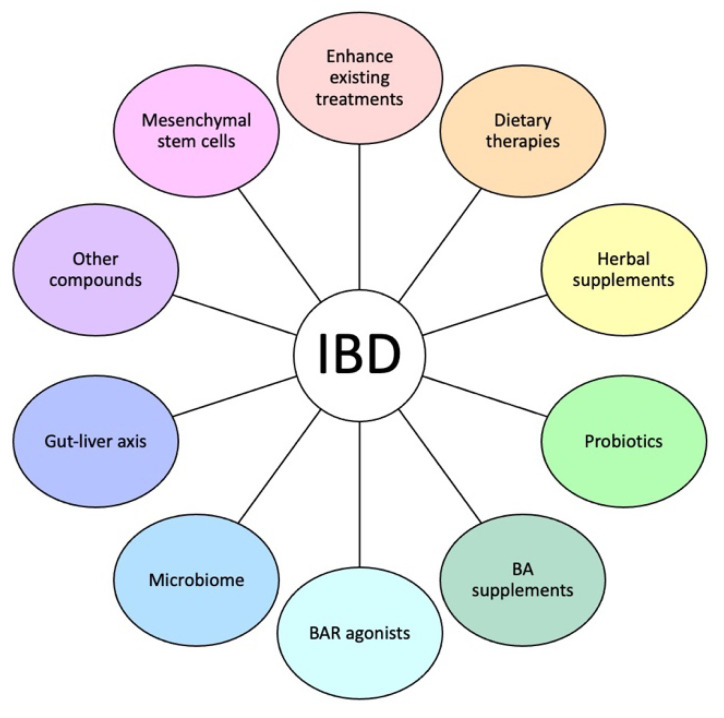
Potential BA-based treatments for IBD. Possible BA-directed therapies to improve IBD treatment include augmenting existing IBD treatments, new dietary treatments, probiotics, BA supplementation, BAR agonists, targeting the gut microbiome and the gut–liver axis, regulating other BA pathways with other compounds, and mesenchymal stem cells. IBD: inflammatory bowel disease; BA: bile acid; BAR: bile acid receptor.

Recent studies have shown that BAs play a role in enhancing the efficacy of existing treatments of IBD, which suggests that BA-based therapies could potentially be effective as both monotherapies and/or used as adjunct therapy with existing IBD treatments. Recent studies have also revealed novel BA-based therapies. These therapies include dietary modifications, herbal medications, probiotics, BA supplementation, BAR agonists, modulators of the microbiota, gut–liver axis, and other BA-mediated pathways, as well as the use of mesenchymal stem cells (Figure 2). In this section, we will highlight current IBD treatments that involve BA-mediated pathways and discuss promising BA-based therapies. 

#### 2.5.1. Enhancement of Existing IBD Treatments

BAs may play a critical role in enhancing the efficacy of existing treatments for IBD [12]. BAs contribute to the therapeutic effects of immunomodulators in IBD treatment. In addition to suppressing TNF, infliximab has been shown to inhibit inflammation by enriching BSH-producing bacteria and restoring BA metabolism. CD patients on infliximab exhibited increased BSH-producing bacteria *Blautia* and *Collinsella*, elevated unconjugated BAs [128], decreased primary BAs [128], and increased FGF19 [129]. BAs are also important in anti-α4β7 integrin therapy. Mice with TNBS-induced colitis who were given an FMT from CD patients who achieved remission with vedolizumab exhibited elevated CDCA and LCA levels, leading to increased stimulation of FXR and TGR5, and ultimately alleviating colitis [122]. The most recent literature suggests that BAs play an important role in augmenting the efficacy of current treatments for IBD. New research exploring the mechanisms of action and interactions between BAs and existing therapies may reveal new treatment strategies for IBD. 

#### 2.5.2. Diets That Modify BAs

The multifaceted role of BAs in IBD has been shown to enhance existing dietary therapies as well as reveal potential new BA-based therapies. BAs have been shown to be key mediators in existing dietary therapies for IBD. Remission in pediatric CD patients by EEN may be mediated by BAs as responders later develop normalization of BA hydrophobicity, which may activate TGR5 receptors and reduce intestinal inflammation [130]. EEN was also found to improve BA dysmetabolism in pediatric CD patients. Enrichment in BAs, including hyocholic acid (HCA) and α-muricholic acid (αMCA), was associated with decreased CD symptoms and increased abundances of *Clostridium innocuum* and *Hungatella hathewayi* [131]. Another study found EEN improved expressions of the Firmicutes phylum and genus Flavonifractor and Clostridium V and upregulation of secondary BAs in pediatric CD patients [132]. In addition to mediating existing dietary therapies, new BA-based therapies include dietary modifications, herbal medications, probiotics, BA supplementation, BAR agonists, modulators of the microbiota, gut–liver axis, and other BA-mediated pathways, as well as the use of mesenchymal stem cells. Recent studies have highlighted several promising dietary therapies for IBD. Western diets exacerbated colitis in pre-clinical models, suggesting that low-fat and low-sugar diets may be beneficial for IBD [105,107,108]. Intermittent fasting ameliorated DSS-induced colitis, partially through decreasing Akkermansia, increasing LCA, and downregulating pro-inflammatory IL-1α, IL-6, keratinocyte-derived chemokine, and the granulocyte colony-stimulating factor [133]. Oral fucose ameliorated DSS-induced colitis by lowering taurine-β-MCA (an FXR antagonist) and TCA, restoring the FXR-FGF15-CYP7A1 pathway, and increasing *Lactobacillus*. Those findings suggest that dietary fucose mitigates colitis by regulating BAs via the gut microbiota [134]. Psyllium improved DSS and T cell transfer-induced colitis by activating FXR and increasing fecal BA [135]. Insoluble dietary fiber alleviated DSS-induced colitis, partly by increasing BA absorption, suppressing the TLR4/NF-KB signaling pathway [136], decreasing *Akkermansia*, increasing *Parasutterella*, *Erysipelatoclostridium*, and *Alistipes*, reversing the DSS-induced decline in secondary BAs, and protecting against DSS-induced intestinal barrier damage [137]. 

#### 2.5.3. Herbal/Natural Supplements

Herbal supplements are part of another potential IBD treatment that has been shown to improve murine colitis by modulating BAs. Several traditional Chinese medicines, including fermented Wallace melon juice [138], Scutellaria baicalensis Georgi [139], Lizhong [140], Huankuile [141], Dahuang-Mudan [142], *Auricularia polytricha* [143], and *Flammulina velutipes* [143], mitigated DSS- and TNBS-induced colitis via BA regulation. CA, CDCA, DCA, and tauroursodeoxycholic acid (TUDCA) were restored to normal levels by *Pleurotus eryngii* [144], *Indigo Naturalis* [145], *Atractylodes macrocephala* Koidz [146], and Sijunzi [147], respectively. Of note, TUDCA is a key component of black bear bile, a traditional Chinese medicine used to treat many inflammatory diseases [148]. Additional studies showed that Si-Ni-San reversed the shift of BA synthesis to the acidic pathway [149], while Gegen Qinlian [150,151] and curcumin enriched the production of secondary BAs [152]. *Rosmarinus officinalis* L. polyphenols increased primary BAs and secondary BAs, which promoted goblet cell proliferation and mucus secretion, thus strengthening the intestinal epithelial barrier [153]. 

Natural supplements have also been shown to alleviate DSS-induced colitis by regulating the gut microbiota-BA-immune axis. *Dendrobium officinale* increased the abundance of Lactobacillales and total BAs [154], while crocetin enriched *Akkermansia* and *Mediterraneibacter* and decreased the levels of primary BAs and secondary BAs [155]. Qingchang Huashi reversed gut dysbiosis and normalized the ratio of deconjugated to conjugated BAs [156]. Alginate promoted *Bifidobacterium animalis* and HDCA synthesis [157]. Apple polyphenol extract decreased HDCA, increased β-MCA, and enriched Verrucomicrobia, Bacteroides, and Akkermansia [158]. Dietary carvacrol and thymol supplementation enhanced the abundance of *Bifidobacterium pseudolongum*, HDCA, and 12-ketodeoxycholic acid, thereby altering the cGMP-PKG-mTORC1 pathway [159]. The grape seed proanthocyanidin improved mild IBD in dogs by enriching anti-inflammatory bacteria CA and LCA [160]. A synthesized metal-phenolic nanozyme with iron and curcumin also enhanced anti-inflammatory gut bacteria as well as LCA and TDCA [161]. *Schistosoma* soluble egg antigen upregulated *Prevotellaceae_UCG-001*, downregulated *Helicobacter*, *Lachnoclostridium*, and *Enterococcus*, and increased BAs [162]. *Sophora alopecuroides* L. increased Firmicutes, Tenericutes, and TM7, decreased Bacteroides and Deferribacteres, and lowered α-, β-, and ω-MCA, CA, transforming growth factor-β1 and IL-1β [163]. N-ethyl-_L_-glutamine from tea increased DCA, CA, and TCA and decreased the MHC-II-dependent presentation of microbiota antigens by the immune system [164]. Gallic acid alleviates colitis by regulating the gut microbiota, increasing ILC3s, and boosting levels of UDCA, isoalloLCA, and 3-oxoLCA [82]. Yiyi Fuzi Baijiant modulated primary BA biosynthesis and the balance of T_H_17/T_reg_ [165]. Herba Origani upregulated Bacteriodota, reversed the decrease in nutriacholic acid, DCA, and HDCA, and downregulated IL-1β and TNF-α to maintain intestinal immune homeostasis [166]. 

Many medicinal plants modulate BA signaling to ameliorate DSS-induced colitis [94]. Fuzhuan brick tea [167], moxibustin [168], and nigakinone [169] regulated BA enterohepatic circulation by activating FXR and inhibiting the TLR4 and NLRP3 pathways. In contrast, sedanolide [170] and trans-anethole decreased FXR by inhibiting the sphingomyelin phosphodiesterase 3 pathway [170] and upregulating FGF15, ASBT, and the bile salt export pump [171]. FXR and TGR5 were upregulated by dihydromyricetin via increased Lactobacillus [172], Akkermansia, CDCA, and LCA. Fucoidan upregulated FXR and TGR5 by increasing CA, UDCA, DCA, and LCA and decreasing β-MCA [173]. Baitouweng Tang normalized BA levels and increased Bacteroidetes, Firmicutes, Proteobacteria, Actinobacteria, Tenericutes, and TM7 [174]. *Eucommia ulmoides* leaves also upregulated TGR5 expression by restoring CA, β-MCA, and DCA to normal levels; increased TCA and TUDCA; and promoted the expression of tight junction proteins [175]. Patrinia villosa improved TNBS-induced colitis by regulating BA metabolism, activating VDR, and inhibiting nuclear factor kappa B (NF-κB) signaling pathways [176]. 

#### 2.5.4. Probiotics 

Probiotics have shown potential as effective therapies for IBD through their regulation of the gut microbiota and BA metabolism. DSS-induced colitis was alleviated by the probiotic strain GR-4 through the upregulation of bacteria that metabolize BAs, by *Bifidobacterium pseudocatenulatum* G7 through an increase in the BA pool size [177], and by *Bifidobacterium longum* (*B. longum*) FGDLZ8M1 by enhancing tolerance to bile salts [178]. *Lactobacillus plantarum* 550 augmented tacrolimus’ improvement in DSS-induced colitis, partially by increasing TCDCA [179]. Probiotics can also exert anti-inflammatory effects by regulating BAs in DSS-induced colitis in mice. The *Lactobacillus casei* strain Shirota increased TCA, TCDCA, TDCA, and TUDCA and decreased α-MCA and β-MCA, inhibiting IFN-γ and upregulated IL-10 via NF-κB suppression [180]. Bacteroides uniformis JCM5828 inhibited T_H_17 differentiation by increasing α-MCA, HDCA, and isoLCA and decreasing UDCA and HDCA [181]. Several probiotic consortiums have been shown to modulate BAs and ameliorate mouse models of colitis. A consortium of *Clostridium* AP sp000509125, Bacteroides ovatus, and Eubacterium limosum, mitigated DSS-induced colitis by restoring secondary BA metabolism to increase UDCA and LCA, which upregulated TGR5 and strengthened the intestinal barrier [182]. An 11-member microbiota consortium GUT-108 reversed colitis in *Il10*^−/−^ mice by increasing LCA and DCA, decreasing colitogenic *Enterobacteriaceae*, and increasing beneficial resident *Clostridium* (Clusters IV and XIVa) species, including *Lachnospiraceae* [183]. A mixture of *Lactobacillus rhamnosus* dm905 and *Lactococcus lactis* mitigated colitis in *Il10*^−/−^ mice, partly by decreasing PXR, TGR5, VDR, CAR, the NLRP3 inflammasome, and pro-inflammatory cytokines [184]. Probiotics may even improve the clinical response to anti-TNF agents. *B. longum* CECT 7894 enhanced the efficacy of infliximab in DSS-mediated colitis by increasing bacteria with BSH and 7α-dehydroxylases genes and upregulating secondary BAs, including LCA, UDCA, and isoLCA [185].

#### 2.5.5. BA Supplements 

BA supplementation is another promising treatment avenue for IBD. UDCA is FDA-approved for cholestatic liver disorders and may also benefit IBD patients by decreasing dysbiosis and mitigating intestinal inflammation. Recent studies showed that UDCA ameliorated TNBS-induced colitis in rats [186] and attenuated colitis in a PSC-IBD mouse model by downregulating mucosal addressin cell adhesion molecule-1 [187]. A dihydroartemisinin-UDCA conjugate also improved DSS-induced colitis by inhibiting T_H_1/T_H_17 differentiation [188]. In a prospective, single-center study, UC patients given UDCA and mesalazine had improved disease compared to the mesalazine-only group, possibly due to upregulation of Firmicutes, downregulation of Proteobacteria, and decreased IL-23 and IL-17 [189]. LCA supplementation reduced intestinal inflammation in DSS−, TNBS−, and CD45RB^hi^ T cell transfer models of colitis via TGR5 [62] and VDR [190], as well as through the mitigation of epithelial cell apoptosis [191]. DSS-induced colitis was also attenuated by administering other BAs. Oral 12-KLCA upregulated VDR and downregulated IL-17A secretion from colonic ILC3s [56]. HDCA potentiated FXR and TGR5 activation [192] and impeded monocyte/macrophage recruitment [193]. HCA decreased TNF-α production by CD4^+^ T cells [131]. A mix of α-MCA and HDCA inhibited T_H_17 differentiation [181]. TUDCA was shown to alleviate inflammation-induced ER stress in intestinal epithelial cells and improve acute and chronic colitis in mice [194]. TUDCA also restored the integrity of the intestinal barrier via claudin-1 and ZO-1 [195]. Recently, oral TUDCA led to significant clinical, endoscopic, and histological improvements in a phase I trial in patients with symptomatic UC by reducing ER stress [196]. Randomized control trials of TUDCA are needed to further evaluate its efficacy in IBD patients. Taurohyodeoxycholic acid alleviated TNBS-induced colitis by restoring the T_H_1/T_H_2 and T_H_17/T_reg_ balance [197]. IsoalloLCA and 3β-hydroxydeoxycholic acid may also have anti-inflammatory properties as they enhance T_reg_ differentiation by inducing the transcription factor forkhead box P3 [198,199]. DCA inhibitors are other potential IBD treatments. Several studies have shown that DCA supplementation worsened mouse models of colitis by inducing gut dysbiosis [200], downregulating the FXR-FGF15 axis [200], increasing intestinal T_H_17 infiltration [201] and the proportion of CD3^+^ and CD4^+^ T cells [202], and decreasing intestinal tuft cells [202]. DCA was also found to delay wound healing through AKT activation, suggesting that AKT inhibitors could be another possible approach for IBD treatment [175]. Improvements to increase the specificity of BA analogs are ongoing. One study designing gut-restricted analogs found that incorporating an N-methyl-D-glucamine group into CDCA increased TGR5 receptor potency with low oral exposure [203]. 

BA supplementation may also improve IBD-associated disease and complications. Many IBD patients also develop PSC. Treatment with UDCA improved PSC in one case study of a pediatric patient with UC and PSC [204]. IBD patients have a higher risk of developing colorectal cancer than the general population. UDCA has been shown to reduce colitis and tumor growth in AOM/DSS-induced colorectal cancer by facilitating *Akkermansia* colonization [186], activating FXR via synergistic effects with intestinal epithelial cells and T_H_17 and ILC3s [205], increasing 3′-phosphoadenosine 5′-phosphosulfate synthase 2 by preventing BA accumulation and increasing the formation of BA sulfates [206], and inducing suppressor of cytokine signaling 1 via TGR5 in macrophages [207]. Patients with PSC-UC have an even greater risk of colon cancer compared to UC patients, which may be due to GDCA inducing microRNA-506 and downregulating p53 [208] as well as due to the increased oncomir microRNA-356 expression downregulating VDR and TNF-α [209]. These studies suggest that targeting BA pathways may be important in preventing colitis-associated colon cancer. Another study found no association between BAs and colorectal cancer in IBD patients, but this study was limited by cohort size as it only had six patients with IBD-associated cancers [210]. The risk of CDI is increased in IBD patients. Pediatric UC patients with CDI had decreased LCA, UDCA, and gut microbial genes for BSH, 7α-hydroxysteroid dehydrogenase, and 7α/β-dehydroxylation compared to those with UC alone, suggesting that BAs could also be potential treatments for CDI in IBD patients [211].

#### 2.5.6. BAR Agonists

In addition to BAs, BAR agonists can be another option for IBD treatment. BARs are one of the primary ways BAs exert their biological effects. BARs are found in a variety of cell types involved in IBD. GPBAR1, FXR, and PXR are highly expressed in innate and adaptive immune systems. Several recent studies have identified various BAR agonists, explored their mechanisms of action, and further elucidated their impact on IBD disease activity. PBT002—a dual GPBAR1 agonist and RORγt inverse agonist—was designed to target both innate and adaptive immunity. GPBAR1 is widely expressed in intestinal epithelial cells, myeloid cells, and NKT cells, while RORγt is expressed in T_H_17, regulatory T cells (T_reg_), and type 3 innate lymphoid cells (ILC3). PBT002 was shown to mitigate disease activity in TNBS−, DSS−, and DSS + IL-23 models of colitis by decreasing pro-inflammatory cytokines [212]. Further, the relative abundance of *Clostridium* spp. (which is reduced by colitis) was reversed in mice treated with PBT002. Given that *Clostridium* spp. is a known BSH-producing bacteria, the results suggest that BAR agonists also play a role in reversing gut dysbiosis associated with IBD. Several other BAR agonists are also described in the recent literature. CAR activation suppressed BA-driven ileitis in a mouse model, suggesting that CAR agonists may be a possible treatment for small bowel CD [213]. Several FXR agonists have shown promise in improving colitis in mouse models. Fexaramine improved DCA-induced intestinal inflammation by restoring FXR activity, activating FGF15, normalizing BA metabolism, and increasing SCFA-producing bacteria [214]. Nelumal A mitigated DSS-induced colitis and AOM/DSS-induced tumorigenesis by increasing FXR expression and tight junctions and decreasing BA synthesis [215]. Moreover, the global FXR agonist obeticholic acid, as well as a PPARα agonist, improved DSS-induced colitis [216]. Interestingly, *Fxr* ablation specifically in the mouse liver ameliorated DSS-induced colitis by enhancing the colon mucus barrier. This may be due to differing regulations of FXR in the liver versus globally [217]. The numerous BAR agonists and their respective mechanisms of action and impacts on IBD disease activity demonstrate the need for future research in this domain. Future research that evaluates the mechanistic pathways of BAR agonists, explores their long-term safety and efficacy, identifies their impact on the gut microbiome, and clarifies their potential interactions with current IBD treatments are necessary. 

#### 2.5.7. Microbiome and the Gut–Liver Axis

Modulation of BA metabolism via the microbiota and the gut–liver axis is another promising strategy for IBD. Patients with IBD lost the microbial *baiCD* regulation of the host angiopoietin-like 4 gene that has been shown to ameliorate DSS-induced colitis [218]. Selectively deleting the *baiH* gene in *Faecalicatena contorta* S122 in a complex microbiome protected against DSS-induced colitis, likely by diminishing Erysipelotrichaceae [212]. These studies suggest that targeting specific microbial genes involved in BA metabolism could be an effective treatment for IBD. Administering microbes with anti-inflammatory properties may be another potential IBD treatment as FMT of Roseburia bacteria in sheep with DSS-induced colitis mitigated intestinal inflammation [219]. FGF19-M52, an engineered FGF19 variant without hepatic pro-mitogenic capacity protected mice from DSS-induced colitis by regulating BA metabolism [220]. Another study showed that deficiency of the Rela/Stat3-CYP enzyme pathway in mouse liver led to a decrease in primary BAs and ameliorated colitis, suggesting that inhibitors of this pathway could offer a novel mechanism to control inflammation in IBD through the gut–liver axis [98]. 

#### 2.5.8. Other BA-Related Therapeutic Targets

Recent studies have identified other potential therapeutic targets of IBD mediated by BAs. Low-dose insulin alleviated DSS-induced colitis in mice by increasing the abundance of *Blautia*, *Enterorhadus*, *Rumi-NK4A214_group*, and LCA, which activated TGR5 and polarization of M1 macrophages [221]. P-glycoprotein was decreased in UC patients, possibly due to lower secondary BA production, and may be a potential UC treatment target [222]. The antimicrobial peptide cecropin A ameliorated DSS-induced colitis, partly by normalizing BA metabolism [217]. Recombinant fibrinogen-like protein 2 improved DSS-induced colitis by reversing the decline in gut microbiota diversity and restoring BA homeostasis by decreasing total BA, increasing FXR and FGF15, and downregulating CYP7A1 [223]. An inhibitor of zinc metallopeptidase glutamate carboxypeptidase II, which was upregulated in IBD patients, was conjugated to DCA and alleviated inflammation in DSS-induced and *Il10^−/−^* models of colitis by lowering intestinal barrier permeability, normalizing tight junction protein expression, and suppressing procaspase-3 activation [224]. Hyperbaric oxygen also showed promise in improving UC by reducing neutrophil STAT3, increasing Firmicutes, decreasing Akkermansia, and increasing LCA [225]. Clinical trials are ongoing to further evaluate the efficacy of hyperbaric oxygen in IBD patients.

#### 2.5.9. Mesenchymal Stem Cells

Mesenchymal stem cells may be another novel therapy for IBD by restoring BA homeostasis [226]. Administering mesenchymal stem cells improved TNBS-induced colitis by increasing gut microbiota α-diversity, enriching Bacteroides, Firmicutes, and Tenericutes, and decreasing Proteobacteria, partially through upregulating secondary BA synthesis [227]. Mesenchymal stem cell-derived exosomes also improved DSS-induced colitis by increasing colonic FXR and improving gut dysbiosis [228]. It remains unclear whether restoring BA levels and composition is required for the efficacy of mesenchymal stem cell transplant. 

Recent studies have enhanced the understanding of BAs and their roles in IBD. To date, approximately 692 different BAs have been discovered, and it is predicted that tens of thousands of new BAs remain to be discovered [77]. Advances in technology and the development of innovative methodologies, including mass spectrometry processes and metabolomics analysis tools, will likely lead to the discovery of new BAs with unique physiological functions and potential therapeutic applications [229,230].

## 3. Conclusions

BAs are critical players in IBD pathogenesis and recent studies have furthered the understanding of the many roles they play in the gut microbiome, immune system, BARs, intestinal epithelium, gut–liver axis, diet, genetics, environment, and other modalities. Studies have demonstrated that patients with IBD exhibit alterations in their BA composition, and both CD and UC patients have generally been found to have an increase in primary BAs and a decrease in secondary BAs. BAs have been found to contribute to intestinal inflammation via several mechanisms, including via BARs, modulation of the immune system via regulation of specific immune cells and inflammatory cytokines, and exerting effects on gut barrier function via regulation of intestinal epithelial tight junctions. The latest research exploring the complex roles BAs play in IBD pathogenesis has revealed several promising targets for therapeutic interventions. Emerging BA-based therapies include new dietary treatments, probiotics, BA supplementation, BAR agonists, mesenchymal stem cells, and targeting the gut microbiome and gut–liver axis. For example, BAR agonist PBT002 has been shown to mitigate disease activity in TNBS−, DSS−, and DSS + IL-23 models of colitis by decreasing pro-inflammatory cytokines [212] and is associated with increased levels of *Clostridium* spp. Diet therapies such as intermittent fasting and oral fructose were shown to decrease DSS-induced colitis via modulating BAs such as LCA and TCA. Probiotic therapies such as *Lactobacillus plantarum* 550 and *Clostridium* AP sp000509125 were shown to improve DSS-induced colitis via the regulation of BAs. Mesenchymal stem cells similarly improved TNBS-induced colitis by influencing the gut microbiota α-diversity partially via upregulation of secondary BAs. BA-directed therapies may be used as standalone treatments or as adjuvants to augment existing therapeutic regimens. Additionally, the altered composition of BAs in patients with IBD shows potential as a promising biomarker for assessing IBD disease activity, as well as predicting and monitoring response to treatment. While the literature reviewed shows promise for new treatment modalities and uses of BAs, there are several limitations. Several of the studies show contradictions in the composition of specific BAs, likely a result of differences in methodologies between studies. Furthermore, while current studies have expanded knowledge of the role of BAs in IBD pathogenesis, especially regarding specific mechanisms of action, it is difficult to ascertain the magnitude of the impact of each individual mechanism of action. Therefore, future studies should define the mechanistic roles of BA-based therapies on a variety of cell types and signaling pathways in the digestive tract and beyond. Controlled clinical trials are also needed to determine their therapeutic potential. Leveraging technological advancements to discover new BAs and enhancing knowledge of the myriad of functions they play in IBD may lead to new classes of therapeutics for IBD that ultimately improve patient outcomes. 

## Figures and Tables

**Table 1:** Alterations in BAs in IBD patients.

Bile Acids	Pooled IBD Samples	Crohn’s Disease	Ulcerative Colitis
**Primary Bile Acids**			
CA		↑ [44,45]	↑ [55,56]
CDCA		↑ [44] ↓ [38]	↑ [56] ↓ [38,64]
GCA	↑ [37]	↑ [46,47]	↑ [56] ↓ [64]
GCDCA		↓ [46]	↑ [55,57] ↓ [64]
GHDCA		↓ [50]	
TCA		↓ [46]	↑ [55,56]
TCDCA			↑ [55,57]
**Secondary Bile Acids**			
DCA	↓ [38]	↑ [47,51] ↓ [48,49]	↓ [50,55,56]
GDCA	↓ [38]	↑ [46,47]	↓ [55]
GLCA	↓ [38]		↓ [55]
HDCA			↓ [56]
isoLCA	↓ [39]	↑ [47]	↓ [56]
12-KLCA			↓ [56]
LCA	↓ [38]	↓ [46,48,49]	↓ [50,55,56,64]
3-oxoLCA	↓ [39]		
TDCA	↓ [38]	↑ [47]	
TLCA	↓ [38]		↓ [55]

## Data Availability

No new data were generated or analyzed in support of this research.
